# Family child care home providers’ perceived difficulty in serving vegetables to children: findings from a multi-method study

**DOI:** 10.1017/jns.2025.9

**Published:** 2025-02-24

**Authors:** Saima Hasnin, Dipti A. Dev, Carly Hillburn, Susan B. Sisson, Alison Tovar

**Affiliations:** 1 Department of Food Science and Human Nutrition, University of Illinois Urbana-Champaign, Urbana, IL 61801, USA; 2 Department of Child, Youth and Family Studies, University of Nebraska–Lincoln, Lincoln, NE 68588-0236, USA; 3 Nebraska Extension, University of Nebraska–Lincoln, Lincoln, NE 68583-0806, USA; 4 Department of Nutritional Sciences, University of Oklahoma Health Sciences, Oklahoma City, OK 73126, USA; 5 Department of Behavioral and Social Sciences, Brown University School of Public Health, Providence, RI 02912, USA

**Keywords:** CACFP, Children, Family Child Care Home, Preschool, Vegetable, CACFP, Child and Adult Care Food Program, FCCH, Family Child Care Home

## Abstract

The study aims to identify family child care home (FCCH) setting- and environment-level predictors related to providers’ perceived difficulty in implementing the Child and Adult Care Food Program (CACFP) recommendations for serving vegetables to children. This was a cross-sectional study, which used a validated paper-based survey with a multi-method data analysis approach. Participants were licenced FCCH providers (N = 943) in Nebraska, who were predominantly White (94%), non-Hispanic (97%), CACFP-participants (89%), and in urban areas (64%). Reflective latent variable modelling was conducted in *Mplus* to explore associations between dependent variable and predictors. Dependent variable was providers’ perceived difficulty to implement CACFP recommendations for serving vegetables. Predictors were providers’ mealtime practices, perceived barriers to serve healthy foods, CACFP participation, geographic location, food access, food insecurity, and child poverty. Qualitative comments (n=122) from the survey were analysed using direct content analysis approach. Providers’ perceived lack of time to prepare foods and perceived children’s taste preferences increased their perceived difficulty; and CACFP-participation decreased their perceived difficulty to implement CACFP recommendations for serving vegetables. Qualitative comments highlighted that providers felt discouraged to serve vegetables knowing that vegetables would likely be wasted because of children’s preferences. More tailored professional development is required to address FCCH providers’ perceived difficulty and build providers’ skills on preparing time saving, CACFP-reimbursable and appealing vegetable recipes, and on strategies to promote vegetable consumption in children.

## Introduction

More than 93% of the children in the United States (U.S.) do not meet the recommendation for daily vegetable consumption.^(1,2)^ Daily vegetable consumption contributes towards prevention of childhood obesity and associated chronic health conditions, including diabetes, cardiovascular diseases, and cancer.^(3)^ However, despite the health benefits, vegetables are the most under-consumed food group among preschool children (3-5-years-old) in the U.S. for over two decades.^(1,4)^ To safeguard the health of young children, the Child and Adult Care Food Program (CACFP) reimburses participating childcare providers to serve nutritious meals daily to more than 4.2 million children (under 5 years) attending the CACFP-participating childcare settings.^(5)^ CACFP requires participating childcare providers to serve children vegetables daily and also recommends preparing vegetables without adding animal fats.^(5)^ Considering the significance of CACFP to support healthy nutrition and thereby preventing childhood obesity, 24 U.S. states have included the CACFP recommendations within their state licensure requirements for all types of licenced childcare settings, irrespective of their programme participation status.^(6,7)^ Moreover, CACFP recommendations are included within the Centers for Disease Control and Prevention (CDC)-high impact obesity preventions standards for childcare and education settings.^(8)^ Because 74% of the 3-5-year-old children in the U.S. attend a form of childcare settings,^(9,10)^ where they consume up to five meals and snacks,^(11–13)^ childcare settings offer an ideal setting to improve children’s vegetable consumption.^(11,14)^ However, previous studies reported limited evidence for effectiveness of CACFP to improve children’s vegetable consumption in childcare settings.^(12,15–17)^

Nationally, there has been a consistent decreasing trend for CACFP-participation rates in family child care home (FCCH) settings.^(18)^ Previous research reported that FCCH providers faced greater challenges in serving meals and snacks according to the updated 2017 CACFP recommendations^(19)^ and demonstrated lower adherence to these guidelines compared to centre-based child care settings.^(20)^ Other studies reported that nutritional quality of the foods served in CACFP-participating FCCH settings can be further improved in terms of the quality of the vegetables served to the children.^(12,17)^ FCCHs are small childcare settings, where typically one provider cares for 6-12 children in their own home and plays multiple roles, such as teaching and supervising children as well as preparing foods and feeding the children.^(21)^ Thereby, FCCH providers have more direct control over the food prepared and served than the centre-based childcare settings and Head Start,^(22)^ giving them an ideal opportunity to positively impact children’s vegetable consumption. Nationally, FCCHs appeal to low-income and rural families because of affordable enrolment fees, flexible hours, and better accessibility than centre-based childcare in rural areas,^(23)^ and currently caring about more than 2.5 million under 5 years old children.^(24)^ However, FCCH settings receive less research attention compared to the centre-based settings, evident by the small numbers of research studies cited in the recently published systematic reviews assessing implementation of nutrition-related practices,^(25)^ nutrition environment,^(26)^ correlates of children’s dietary intake,^(27)^ and nutrition interventions^(28)^ at childcare settings.

Limited number of research studies have documented factors that are associated with nutritional quality of the foods served in FCCH settings but not specifically regarding the vegetables served. Such as FCCH nutrition policy;^(12)^ providers’ feeding practices for example role modelling and eating the same foods with the children;^(29)^ and CACFP-participation^(30)^ were positively associated with higher nutrition quality of the foods served. Again, providers’ perceived barriers were associated with lower nutrition quality of foods served in FCCH. These barriers included children’s taste preferences, limited time for food preparation, and insufficient funds to purchase fresh produce^(31–33)^. In addition to the above-mentioned factors occurring within the FCCH setting, broader environment-level factors like FCCH geographical location (urban/rural), food access, food insecurity, and child poverty may also influence nutritional quality of the foods served in FCCH. Specifically, Speirs et al. (2020) reported rural FCCH providers had higher perceived difficulty meeting CACFP recommendations compared to urban providers^(19)^. Further, FCCH providers with lower neighbourhood food access reported more barriers to serving healthy foods to children than providers with higher access^(34)^. Neighbourhood food access^(35)^ and poverty^(36)^ were also related to increased household food insecurity and poor diet quality in children. However, it is unknown how these environment-level factors are related to children’s nutrition in FCCH settings.

Taken together, we have an initial understanding of factors that are related to nutritional quality of the foods served to children in FCCH. However, suboptimal implementation of the recommended CACFP practices at FCCHs^(12,26,37)^ indicate that there is a need for further research on providers’ perceived difficulty in implementation of CACFP recommendation, especially for serving vegetables to the children. Perceived difficulty is defined as ‘how difficult it is to implement the recommended practices’.^(38)^ Based on the Consolidated Framework for Implementation Research, perceived difficulty affects the quality and rate of implementation of recommended practices.^(38)^ This means FCCH providers’ perceived difficulty may influence the quality and rate for providers’ implementation of CACFP recommended practices regarding serving vegetables.

We hypothesised that FCCH setting-level predictors, such as provider’s participation in CACFP,^(39)^ role modelling,^(27,29,40)^ eating the same foods with the children,^(27,29)^ and fewer perceived barriers to serve healthy foods to children^(25)^ would be associated with lower perceived difficulty to implement CACFP recommendations for serving vegetables. We also hypothesised that environment-level predictors, such as providers’ rural geographical location,^(41)^ low local food access, food insecurity, and child poverty would be associated with higher perceived difficulty to implement CACFP recommendations for serving vegetables.

## Methods

### Study design

This was a multi-method exploratory cross-sectional study using a state-wide representative data set. In this research study, prediction and predictors refer to statistical prediction and do not imply causal relationships. The present study considers both multivariate and multi-method analyses to determine predictors for providers’ perceived difficulty to implement CACFP recommendations for serving vegetables in FCCH. The multivariate structural equation modelling approach offered three major advantages over a multivariate regression model: (a) explicit assessment of measurement error for both independent and dependent variables, (b) estimation of latent (unobserved) variable via two dependent (observed) variables in a single statistical model, and (c) the developed theoretical model could be evaluated for fit of the sample data. Additionally, given that FCCH, especially rural FCCH, is under-represented in the literature,^(25–27,42)^ the multi-method approach offered the following two advantages over single methods: provides an exhaustive list of barriers and provides researchers the chance of an indirect check of desirability bias through exploring the consistency between quantitative evaluations and qualitative interpretations of FCCH providers’ perceived barriers. The study was approved and exempted by the Institutional Review Board of University of Nebraska-Lincoln.

### Settings and participant recruitment

A list of all licenced childcare providers was retrieved from the website of the Nebraska Department of Health and Human Services. In January 2017, a paper-based survey, cover letter outlining the study objectives, $1 cash incentive, and prepaid return envelope were mailed to 3,014 licenced childcare providers in Nebraska. Between March and April 2017, non-responders received a reminder postcard, followed three weeks later by a second survey packet without the incentive. The response rate for the survey was 54.6%. In the current study, we only considered the FCCH subset (n= 970) caring for 3-5-year-old children. We excluded participants responding that they cared for >12 children in their setting per day, as they could not be classified as FCCHs based on the definition for licenced FCCH setting in Nebraska. Additionally, we removed participants who had missing responses for their programme location’s zip code, resulting in a total sample size of 943.

### Quantitative data collection

#### Survey

The data for the 86-item survey were collected through a surface mail service using a paper-pencil modality.^(22,43)^ Items were drawn from a previously published and validated survey.^(33,44,45)^ Specifically, the nutrition-related best practices were drawn from Benjamin et al.^(46)^ and questions regarding barriers were drawn from Whitaker et al.^(45)^ During the development phase an interdisciplinary advisory committee reviewed all survey items, and cognitive testing was conducted with two FCCH providers to ensure face validity.^(22,43)^

#### Analytical variables

##### Outcome variable

FCCH providers’ perceived difficulty to implement CACFP recommendations for serving vegetables was the latent variable with the following two indicator (dependent) variables, measured using a 4-point Likert scale [*where 1= Not at all difficult, ….4= Very difficult*].

(1) how difficult it is to serve vegetables at least one time per day (please do not include French fries, tater tots, hash browns or dried beans); and

(2) how difficult it is to prepare cooked vegetables without adding meat fat, margarine, lard, or butter.

Thus, higher perceived difficulty for serving vegetables daily and for preparing vegetables without adding animal fats would indicate higher perceived difficulty in meeting CACFP recommendations to serve vegetables, which was the outcome variable (latent variable).

##### Predictor variables

Respondents self-reported their age, sex, race and ethnicities, education, work experiences, adherence to nutrition-related best practices, mealtime feeding practices, nutrition education, family engagement, preferences for professional development, and barriers related to serving healthy foods and beverages using dichotomous responses (*‘Yes’* and *‘No’*).

FCCH setting-level predictors (n=9) included providers’ participation in CACFP, frequency of receiving professional development training, two mealtime practices, and five perceived barriers. Two evidence-based mealtime practices, which were directly associated with children’s vegetable consumption in childcare in previous studies^(27,29,40,47,48)^ were included as FCCH setting-level predictors: *Providers eat only the food and beverages that are being served to children during meals and snacks* and *providers enthusiastically role model eating healthy foods served at meal and snack times.* The survey originally had 13 items for providers’ perceived barriers to serve healthy foods and beverages to the children. The current analyses considered five of the 13 items, which were perceived as a barrier for at least 20% of the participants; the other eight items were not included in the current analyses. The barriers we included in the current analyses were: *So many different recommendations that providers do not know which to follow; weekly schedule limits time to shop more than once per week; not enough money to cover the cost of serving healthier meals and snacks; those preparing meals and snacks lack the time to prepare healthier foods and beverages; and children would not like the taste of healthier meals and snacks.*

Environment-level predictors (n=4) included geographic location, neighbourhood food access, food insecurity, and child poverty. Participants’ geographic location (urban/ rural) status was determined using the Rural-Urban Continuum Codes (RUCC) published in 2013, where counties scored 4-9 were considered rural^(49)^. Food access, food insecurity and child poverty were coded using geographic identifiers (GEOIDs) derived from the National Historical Geographic Information System-Census Tracts^(50)^ using participant zip codes. These GEOIDs were used to merge the dataset with publicly available national and state-level census tract data to determine each FCCH provider’s neighbourhood food access,^(51)^ food insecurity,^(52)^ and child poverty scores.^(53)^ Low *food access* was characterised by at least 500 people and/or 33% of the tract population residing >1 mile from a supermarket or large grocery in urban areas, and >10 miles in rural areas.^(51)^ *Food insecurity* was defined as the percentage of food-insecure individuals living in households with specific income ranges.^(52)^ *Child poverty* was defined as the percentage of people (<18 years) in poverty in that area.^(53)^

### Qualitative data collection

The qualitative data source in the current study was the self-report survey as well. In addition to the questions with dichotomous options, the survey asked the following open-ended question *‘Please describe any other barriers not listed above’* relating to serving foods and beverages to the children. In total, 122 FCCH providers (12.6% of the total sample) responded to this open-ended question. These 122 sets of qualitative comments ranged in length from a few words to multiple sentences indicating providers’ perceived barriers. No additional recruitment and sampling occurred for the qualitative data collection. Additionally, respondents were not contacted for follow-up interviews.

### Data analysis

Data analyses involved a joint analysis of quantitative data and qualitative comment data from the self-reported survey.

#### Quantitative data analysis

We used SPSS (version 27.0) for descriptive statistics, dummy coding the categorical variables, coding missing variables as ‘999’, and to explore bivariate correlations between the variables (Supplementary Table S1). For multivariate analysis, reflective latent variable modelling was performed using Mplus (version 8.0)^(54)^ to explore the association between the perceived difficulty to meet CACFP recommendations to serve vegetables and literature supported predictors[Table tbl2]. We ran two different versions of the reflective latent variable model. Version 1 included all predictor variables. [Table tbl2]However, as model version 1 did not converge we ran model version 2 excluding food access, food insecurity, and child poverty as predictors. This is also supported by *a priori* bivariate analyses. Based on the bivariate correlation analyses, the three geo-coded environment-level predictors (food access, food insecurity, and child poverty) in Supplementary Table S1 (6^th^, 7^th^, and 8^th^ rows) were correlated with each other but were not correlated with the providers’ perceived difficulty to serve vegetables, i.e., the dependent indicator variables. Consequently, only model version 2 has been reported and discussed in the results. Due to incomplete survey responses, 2.4% of the data were missing. However, MPlus uses all available information for the model development and does not perform listwise deletion. Additionally, Maximum Likelihood Estimation with Robust Standard Errors (MLR) was used to address non-normality and missingness of the data. The covariance coverage for the descriptive statistic output ranged from .231 to .974. Multiple indices were used to assess global model fit: the Comparative Fit Index (CFI>.90), Tucker-Lewis Index (TLI>.90), Root Mean Square Error of Approximation (RMSEA<.08), and Standard Root Mean Residual (SRMR<.08).

#### Qualitative data analysis

For qualitative data analysis and reporting COREQ (Consolidated Criteria for Reporting Qualitative Studies) checklist was followed.^(55)^ To achieve acceptable levels of reliability on how comments were coded, we used a multistep coding process– segmentation of text, codebook creation, initial independent coding, assessment of coders’ reliability, codebook modification, and final coding.^(56,57)^ Two initial coders (S.H. and C.H.) trained in qualitive research first familiarised themselves with the data by thoroughly reading the comments multiple times until the content became familiar and had initial understanding of the patterns.^(58)^ While doing so, the coders found that most of the providers wrote about barriers that were already listed as closed-question statements in the survey, but providers gave more explanations sharing the thought processes behind their perception. The open-ended survey comments were entered into an Excel spreadsheet and were coded manually to divide 2 global themes following the levels for predictors in the quantitative statistical model: perceived barriers at the (a) FCCH setting-level and (b) environment-level. This was followed by categorising the comments into 8 subthemes reflecting to the barriers listed as closed question statements in the self-report survey. Comments that included multiple subthemes were placed into more than one category.

A final table was produced to capture the themes, subthemes, and representative comments. The inter-coder agreement between the two initial coders was high (95%). Finally, the table was checked by another researcher (D.A.D) experienced in qualitative research methods and relevant field expert to assess the consistency between the data presented and themes and subthemes. The two coders and relevant field expert discussed any inconsistencies until a verbal consensus was reached during a debriefing meeting. The participants were not asked to review the final themes, subthemes, and example comments.

## Results

The current study included 943 FCCH providers’ responses, with 600 providers from urban and 343 providers from rural Nebraska, representing 45% of the total number (n = 2151) of FCCH settings in 2017.^(59)^ Providers’ demographic characteristics, programme-level variables, and providers’ feeding practices are presented in Table 1. The majority of the FCCH providers were non-Hispanic White (94.2%) and CACFP participants (89%). On average, number of White children attending the FCCH programmes were 7.7 (±3.2) and number of Hispanic children attending the programmes were .4 (±1.1).

**Table 1.** Demographic characteristics of the Family Childcare Home (FCCH) providers in Nebraska and descriptive statistics (N=943)

Characteristics	Mean ± SD or N (%)
*Environment level*	
Location of FCCH*^a^*	
Urban	600 (63.6%)
Rural	343 (36.4%)
Food insecurity^b^	11.8 ± 1.8
Food access^c^	6.0 ± 4.7
Child poverty^d^	13.8 ± 3.6
*FCCH setting level*	
Provider’s age (in years)	48.5 ± 11.9
Provider to child ratio	1 provider to 8.6 children
Children’s race for each FCCH programme (on average)	
American Indian or Alaskan Native	.3 (2.8%)
Asian	.2 (1.9%)
Black	.9 (8.5%)
Native Hawaiian or Pacific Islander	.1 (.9%)
White or Caucasian	7.7 (72.6%)
Mixed	1.1 (10.4%)
Others	.3 (2.8%)
Hispanic children attending at each FCCH programme (on average)	.4 (3.8%)
Provider’s race	
White	888 (94.2%)
Black	21 (2.2%)
Other and Mixed	34 (3.6%)
Provider’s Ethnicity (Hispanic/Latino/x/a)	24 (2.6%)
Provider’s Education	
High School	283 (30%)
Some College	260 (27.6%)
2-Year Degree or Higher	345 (36.6%)
Participation in CACFP	
Yes	838 (89%)
No	72 (7.6%)
Provider’s feeding practices	
Role modelling	
Yes	827 (90.1%)
No	91 (9.9%)
Eat the same food	
Yes	540 (59.9%)
No	361 (40.1%)
Provider’s perceived barriers	
Too many recommendations	
Yes	186 (19.7%)
No	734 (77.8%)
Lack of time to shop	
Yes	353 (37.4%)
No	569 (60.3%)
Lack of money	
Yes	406 (43.1%)
No	517 (54.8%)
Lack of time to prepare	
Yes	211 (22.4%)
No	702 (74.4%)
Children’s taste preferences	
Yes	346 (36.7%)
No	569 (60.3%)
Frequency of professional development	
Never	69 (7.5%)
Less than once/year	134 (14.6%)
At least once/year	336 (36.7%)
2-3 times or more/ year	376 (41.1%)
Providers’ perceived difficulty	
To serve vegetable once a day	
Not at all difficult	805 (85.4%)
A little difficult	65 (6.9%)
Kind of difficult	21 (2.2%)
Very difficult	10 (1.1%)
To prepare vegetable without fat	
Not at all difficult	69 (7.3%)
A little difficult	134 (14.2%)
Kind of difficult	336 (35.6%)
Very difficult	376 (39.9%)

a Geographic location (urban/rural) status was determined using the Rural-Urban Continuum Codes (RUCC) published in 2013,^(49)^ where counties scored 4-9 were considered rural.

b Low *food access* was characterised by at least 500 people and/or 33% of the tract population residing >1 mile from a supermarket or large grocery in urban areas, and >10 miles in rural areas.^(51)^

c *Food insecurity* was defined as the percentage of food insecure individuals living in households with specific income ranges.^(51)^

d *Child poverty* was defined as the percentage of people (<18 years) in poverty in that area.^(52)^

### Quantitative results

Supplementary Table S1 reports the bivariate correlation values across the variables considered in the current study analyses. The outcome variables: *perceived difficulty to serve vegetables once a day* and *prepare vegetables without fat* were positively correlated with each other (*r* = .91, *p*<.001). These two outcome variables were significantly correlated with FCCH setting-level predictors but not correlated with any environment-level predictors. Providers’ perceived barriers, such as *having too many recommendations* (*r* = .07, *p*=.02), *lack of time to shop* (*r* = .08, *p*=.01) and *prepare healthy foods* (*r* = .12, *p*<.001) were correlated with the outcome variable *perceived difficulty to serve vegetable once a day*. Again, providers’ perceived barriers such as *lack of time to shop* (*r* = .07, *p*=.03) and *prepare healthy foods* (*r* = .11, *p*<.001) were correlated with the outcome variable *perceived difficulty to prepare vegetables without fat*.

**Fig. 1.**
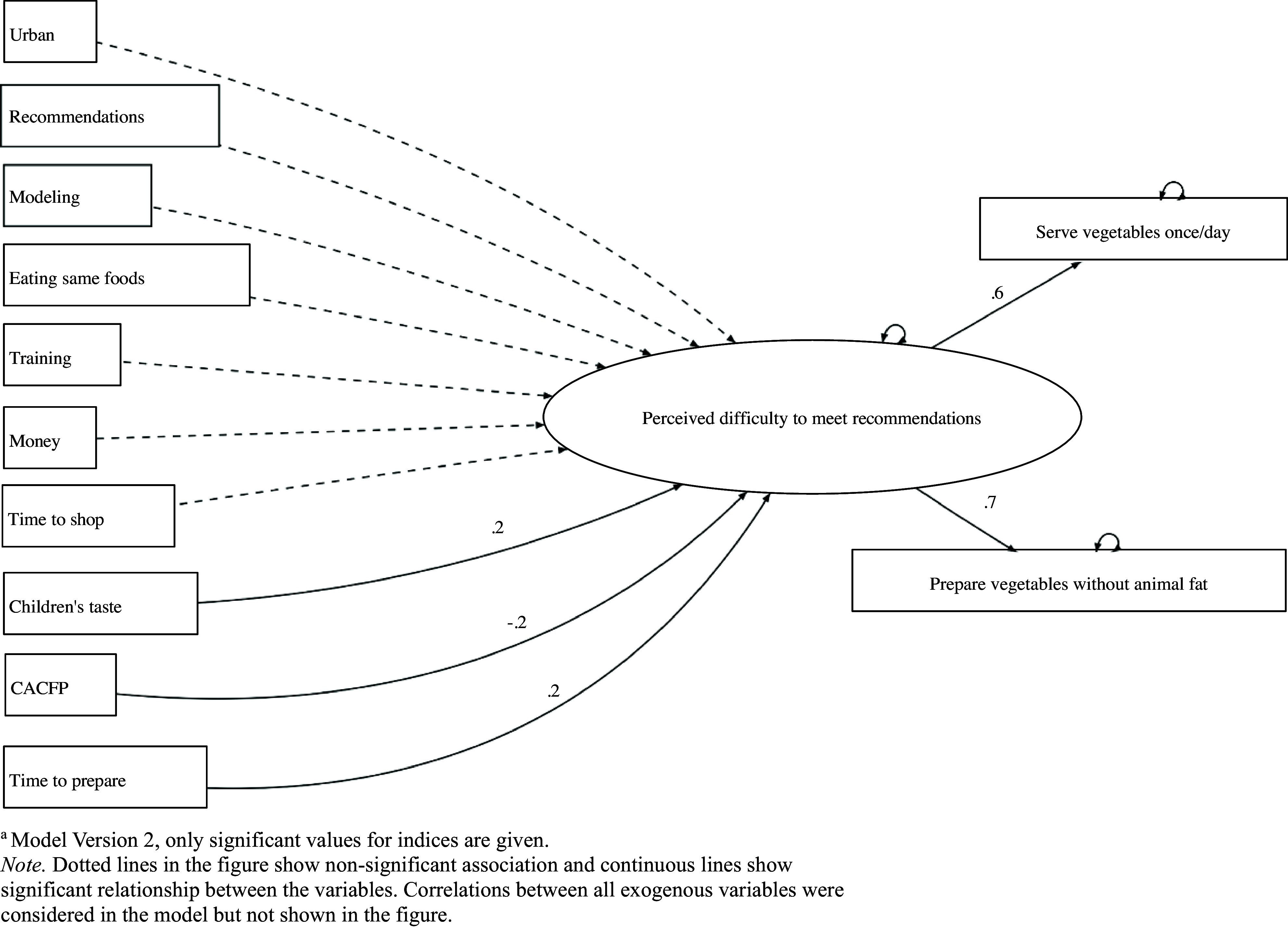
Reflective Latent Variable Modeling for Identifying Predictors of Family Child Care Home Providers’ Perceived Difficulty to Implement CACFP Recommendations for Serving Vegetables to Children.

For the multivariate analyses, the output for latent variable model version 2 yielded good global fit indices, where food insecurity, food access, and child poverty were not included (Table 2 and Fig. 1). Multiple indices were considered to determine the model fit, such as the chi-square, χ^2^ = 5.1 (*p* =.82), CFI = 1.0, TLI = 1.0, RMSEA = 0, SRMR = 0.01.

**Table 2. tbl2:** Literature-supported factors relating to the adherence to best practices for serving vegetables to children in Family Childcare Home (FCCH) settings in Nebraska (N= 943)^a^

Predictors	Standardised Estimates *(SE, p-value)*
Environment-level	
Geographical location of the programme is rural	−.02, .69
FCCH setting-level	
Participation in Child and Adult Care Food Program (CACFP)	−.19, .01^*^
Frequency of providers receiving professional development (Training)	−.05, .32
*Providers’ practices*	
Eating same foods with the children	−.02, .74
Enthusiastically role model	.09, .05
*Providers’ perceived barriers*	
Too many recommendations	.1, .1
Lack of time to shop	. 04, .46
Lack of money	−.03, .61
Time to prepare foods	. 22, <.001^**^
Children’s taste preferences for healthy foods	.18, <.001^**^

a The Table 2 only shows statistics from the reflective latent variable model version 2 ran in MPlus.^(54)^ This version of the model did not include food insecurity, food access, and child poverty as predictors.

* Indicates statistically significant difference at *p* <.05.

** Indicates statistically significant difference at *p* <.001.

The two outcome variables: perceived difficulty to serve vegetables at least once a day (*b*= .313, p<.001) and prepare vegetables without fat (*b*= .459, *p* <.001) were positively associated with the latent variable– ‘*perceived difficulty to implement CACFP recommendations for serving vegetables’*. Table 2 left column shows all the predictors (n=10) considered in the final multivariate model, in which three FCCH setting-level predictors significantly predicted the latent variable. Results showed that CACFP participating FCCH providers had about 19% (*b=* -.19, *p=*.011) lower perceived difficulty to implement CACFP recommendations for serving vegetables compared to non-CACFP participating providers. Providers’ lack of time to prepare healthy foods increased providers’ perceived difficulty to meet CACFP recommendations to serve vegetables by 22% (*b=*.22, *p* <.001). Children’s taste preferences increased providers’ perceived difficulty to meet CACFP recommendations to serve vegetables by 18% (*b=*.18, *p* <.001).

*Rural geographical location* of the FCCH programme was not significantly related to the latent variable. Overall, the combination of the FCCH setting-level and environment-level predictors explained 18.7% of the variance in providers’ perceived difficulty to implement CACFP recommendations for serving vegetables, suggesting a small effect size (.18).

### Qualitative results

In total, 122 set of comments were categorised into two global themes: providers’ perceived barriers (a) at the FCCH setting-level and (b) at the environment-level; under which eight subthemes emerged.

***(a) Providers’ Perceived Barriers at the FCCH Setting Level***

At the FCCH setting-level providers’ perceived barriers included– Lack of family support (n=33), High plate waste of vegetables owing to children’s eating behaviours (e.g., preferences, picky eating, and food neophobia) (n=31), Disagreement with healthy food recommendations (n=14), Lack of time to prepare healthy foods (n=23), Lack of knowledge (n=6), and Others (n=14).

**Lack of family support.** Providers highlighted that it is hard for them to communicate with families. FCCH providers felt that although the families support healthy food plans at childcare, families provide sugary foods and snacks to the children when at home and for celebrations. Providers mentioned that children eat ‘non-healthy’ foods at home, are already full when parents drop their children at the childcare settings, and children do not learn to eat healthy at home which makes it harder for providers to encourage children to eat healthy at childcare. For example, ‘Kids are used to getting non-healthy (unhealthy) food at home’. Providers also emphasised educating families to promote healthy eating in children, such as ‘Parents need to be educated on healthy foods for their children’.

**High plate waste of vegetables owing to children’s eating behaviours (e.g., preferences, picky eating, and food neophobia).** FCCH providers perceived that children under their care do not like the taste of vegetables. Additionally, some providers perceived the children attending their settings were picky-eaters and have food neophobia. Thus, children usually waste the healthy foods owing to lack of preferences, which increased providers’ perceived difficulty as the healthy foods were already expensive for them to serve. For example,

It’s the kids in general. They refuse to eat vegetables certain fruits and meats. We are required to offer/serve it and just goes to waste because they don’t eat it at home. So, they don’t eat it at daycare… so frustrating.

Other providers mentioned ‘Mostly lack of money to spend on fresh fruits & veggies when the kids will not eat it. I hate wasting food!’ and ‘I’ve wasted a lot of healthy, expensive food because the children do not like it’.

**Disagreement with healthy food recommendations.** CACFP recommends serving vegetables during snacks and requires providers to serve low or fat-free, and no sugar-added grain, meat, or dairy products. Providers stated that they thought the CACFP recommendations are too strict, and it is hard for them to keep up with the changes in the recommendations. Providers found it difficult to follow recommendations to restrict sugar and fat in breakfast, snacks, and milk and doubted that this is a viable way for promoting health in children. One provider stated– ‘The fact that snacks are changing as well really stinks for our kids– it’s not the food making our society fat. It’s the lack of movement and the ease of technology-!!’ Providers also shared that ‘…they [children] should just be allowed a cookie’. Another provider quoted– ‘My personal opinion is the rules are getting ridiculous… common sense should be used. Better to have them eat some, than to waste as much as they do!’

**Lack of time to prepare healthy foods.** FCCH providers also shared that they found it very hard to prepare and serve healthy foods while attending to the children. As one provider mentioned ‘For me financial (and) time are most of the issue. Prep-time (away) from the kids, I already work 12 hours so doing outside of care only expands that’. Additionally, as healthy meals are generally wasted discouraged the providers to spend time preparing, such as- ‘Having the time to cook 5 healthy meals per day is very difficult. The kids actually eating these healthy meals is very difficult. They end up wasting sooo much healthy expensive food’.

**Lack of knowledge about healthy food preparation.** Childcare providers stated that not enough information is available regarding what healthy foods are and how to prepare healthy foods in an appealing way. For example, several providers thought that healthy foods are organically grown produce and are expensive. Providers shared the ‘need for more recipe ideas to make healthier foods more appetising to kids who only eat junk food at home’. Providers also shared that they ran out of ideas for serving healthy foods with different recipes to the children around the week. Many providers noted struggling to find enough variety in their meal planning. Providers mentioned:

Children do not like me to serve same food multiple times a week so it is a waste to buy fresh fruit, veggies if I can’t feed it multiple days in a week…. the number of times an item may be served in a week even if it is used differently each time (is a barrier).

**Others.** A few providers shared their self-reflection stating that they already serve healthy foods to the children, and they would like to continue trying to serve healthy foods in the future. Other providers mentioned barriers regarding their struggles to serve children who have food allergies or are vegan but underweight.

**(b)** ***Providers’ Perceived Barriers at the Environment Level***

Subthemes for providers’ perceived barriers at the environment-level were cost of healthy foods (n=36) and limited varieties of healthy foods available in nearby grocery stores (n=17).

**Cost of healthy foods.** Providers perceived that the reimbursement rate from the federal programme is not enough to cover the cost of healthy foods for all the meals they serve to the children. Providers mentioned that ‘Not enough reimbursement to buy very much healthier meats and vegs (vegetables) & fruit– fresh not canned’. Providers also added that serving healthy foods cut their profits from their business. For example, one provider quoted ‘Food programme (CACFP) does not pay that much for meals so most time it comes out of pocket to try and feed healthier’. Similarly, another provider noted, ‘Healthy food is expensive. Cuts into slim profit marg(in)’.

**Limited varieties of healthy foods available in nearby grocery stores**. Providers perceived that they have low availability of fresh foods and limited variety in the nearby grocery stores. A few providers have pointed out that big supermarket stores are 20-70 miles away, and it is hard for them to access fresh produce from these stores weekly.

In summary, based on the qualitative findings FCCH providers felt discouraged to serve vegetables with their limited time to prepare and shop for healthy foods because vegetables were likely to be wasted. Additionally, since vegetables were costly and CACFP reimbursements were low, implementing CACFP recommendations caused them to lose their business profits. Further, providers reported that children had low taste preferences for vegetables, many children were picky eaters, had food neophobia and did not eat vegetables at home. These factors contributed to the high plate waste of vegetables.

## Discussion

The present study provides unique insights regarding FCCH **providers’** perceived difficulty in implementing CACFP recommendations for serving vegetables to children, underscoring the need to better understand the FCCH organisational structure and creating targeted education materials and training opportunities for the FCCH providers.

Children’s taste preferences and lack of time to prepare foods were the two barriers that increased providers’ perceived difficulty to implement CACFP recommendations to serve vegetables, which was the outcome variable (latent variable). These findings align with a previous quantitative study conducted by Patel and Colleagues (2022) exploring providers’ low adherence to CACFP recommendations.^(31)^ Specifically, Patel et al. (2022) reported that FCCH providers who were concerned about food waste due to child’s taste preferences were less likely to meet CACFP recommendations for serving vegetables.^(31)^ Providers’ low adherence to the reimbursement owing to their concerns about children’s taste preferences and associated plate waste of vegetables warranted further justification. The qualitative results in the current study provided this justification and advanced our understanding regarding FCCH providers’ perceived difficulty to serve vegetables. Because FCCH providers are responsible for multiple roles while simultaneously taking care of the children of mixed ages and manage their business, they have limited time and resources available to shop, prepare and serve meals using healthy food options and a variety of recipes.^(60)^ Thus, with limited profit margins providers in this study felt demotivated to continue serving vegetables to the children, knowing that it might go to waste.

These findings emphasise the need for more targeted professional development catering to the FCCH’s unique organisational structure on how to promote vegetable consumption in preschool children. Previous experimental studies in centre-based settings show that evidence-based vegetable preparation practices, such as incorporating vegetables within entrée, serving nutritional dips with the vegetables, adding salt during preparation, and serving vegetables before other foods are associated with children’s vegetable intake.^(61–63)^ Moreover, other evidence-based strategies, such as– repeated exposure, interactive shared book reading, and sensory exploration improve children’s taste preferences for vegetables, willingness to try new and previously disliked vegetables, and consumption.^(64–67)^ However, such interventions were not adapted or evaluated for FCCH settings. Therefore, future research may explore the feasibility and efficacy of these evidence-based strategies to improve children’s taste preferences and vegetable consumption in FCCH settings and thereby, reduce plate waste.

The current multi-method, multivariate study also found that FCCH setting-level predictors were significantly related, while environment-level predictors were not related to meet CACFP recommendations for serving vegetables. This contrasts with the findings from Speirs et al. (2020) using bivariate statistical model to explain that FCCH providers in rural areas perceived higher difficulty to meet CACFP recommendations for preparing meals than urban providers.^(19)^ This discrepancy again indicates that the FCCH organisational structure and related barriers for serving vegetables to children go beyond the environmental-level factors and highlight on the need for more individualised behavioural education intervention along with policy, system, and environmental (PSE) changes.

The qualitative findings in the current study shed additional light on the FCCH providers’ unique barriers that did not emerge in previous research. Low food access in rural areas is a frequently cited barrier in research.^(68)^ However, this perspective often overlooks the critical issue of variety. While nutritional recommendations and CACFP standards emphasise the importance of serving a variety of vegetables,^(5,69)^ rural areas often face challenges in both the quantity and diversity of available options.^(70)^ Providers in our study were also concerned about spoilage of the bulk purchased produce. Because FCCH providers have a lack of time to grocery shop they save time by purchasing groceries in bulk.^(60,71)^ Our findings added that providers need more recipe ideas to use the bulk-purchased foods in a variety of ways around the week to avoid food spoilage. Additionally, FCCH providers in the current study also held the misperception that only organically grown vegetables are healthy and that all healthy foods are generally expensive. However, in practice, even canned and conventionally grown vegetables are considered healthy options that are cheaper and have more shelf life compared to the fresh produces.^(72)^ Finally, providers perceived that although families support the idea of healthy eating, they do not serve healthy foods to children at home and send unhealthy snacks with the children to the childcare. Thus, nutrition educators and CACFP sponsoring organisations are suggested to offer more training opportunities for FCCH providers on how to create varieties of child-approved recipes using similar fresh ingredients around the week, how to preserve bulk-purchased fresh produce within limited kitchen space, and how to engage families to promote vegetable consumption in young children.

### Limitations and strengths

The study findings should be interpreted while considering the following limitations. First, the findings rely on FCCH providers’ self-reported barriers and challenges in meeting CACFP vegetable-serving recommendations. As both predictors and outcomes are self-reported and survey-based rather than observed, this may introduce social desirability bias and is not suitable for causal inferences. Second, the data were collected in early 2017, thus may not reflect the most recent perceptions of FCCH providers because the COVID-19 pandemic has increased FCCH providers’ barriers, as evidenced by higher CACFP drop-out and business closures.^(73)^ The pandemic has also exacerbated the condition for early childhood obesity.^(74)^ However, the present study findings are supporting or expanding on research published after COVID-19 suggesting the relevance of the findings.^(31,32,71)^ Third, the survey asked providers’ perceived barriers regarding serving healthy foods and not specifically about barriers related to serving vegetables. Fourth, the study was conducted only with FCCH providers in Nebraska limiting the generalizability to other U.S. states. Hence, future research should consider exploring similar research questions in other U.S. states with a more racially and ethnically diverse population.

Despite the above-mentioned limitations, the methods and data analysis plan of the current study was rigorous. The use of multi-method data analysis approach allowed us to employ data triangulation to improve the overall quality, authenticity, and trustworthiness of the evidence presented. Specifically, outcome variable was specific to CACFP recommendations for serving vegetables. Additionally, vegetables are reported as the least consumed healthy food group in the childcare settings,^(2,17)^ costly, less available in fresh condition, and were very commonly mentioned in the qualitative comments in our study. Other strengths include the large sample size (45% of total FCCH in Nebraska),^(75)^ with representation and diversity based on FCCH geographic location. Finally, during survey dissemination, the providers were ensured that the data will be analysed and reported in a group format to reduce social desirability bias.

### Conclusions

This study highlighted the key barriers that FCCH providers face in meeting CACFP vegetable recommendations: high plate waste, lack of vegetable variety and recipe ideas, and children’s preferences for vegetables. Particularly, the unique organisational structure of FCCH settings, where providers manage multiple roles, including child care and business operations, may contribute to these challenges of time constraints and limited resources for meal planning. The findings underscore the need for targeted professional development that equips providers with practical strategies for overcoming these barriers, such as evidence-based vegetable preparation practices and ways to engage families in promoting healthy eating at home. Future research should explore the feasibility and effectiveness of these strategies in FCCH settings to improve vegetable intake and reduce plate waste, with a focus on overcoming logistical and organisational challenges in FCCH.

## Supporting information

Hasnin et al. supplementary material Hasnin et al. supplementary material

## Supplementary material

The supplementary material for this article can be found at https://doi.org/10.1017/jns.2025.9.
