# Supplementary material for: Family child care home providers’ perceived difficulty in serving vegetables to children: findings from a multi-method study
Source: J Nutr Sci. 2025 Feb 24;14:e21. doi: 10.1017/jns.2025.9 (PMC11867830; doi:10.1017/jns.2025.9)
Supplement: Hasnin et al. supplementary material [file S2048679025000096sup001.docx]

| **Supplementary Table S1**  ***Bivariate Correlation across in (blinded for peer review) FCCH Setting-level and Environment-level Predictor Variables and Providers’ Perceived Difficulty to Implement CACFP^a^ Recommendations to Serve Vegetables to Children in 2017 (N= 943).*** | | | | | | | | | | | | | | | |
| --- | --- | --- | --- | --- | --- | --- | --- | --- | --- | --- | --- | --- | --- | --- | --- |
| Variables | 1 | 2 | 3 | 4 | 5 | 6 | 7 | 8 | 9 | 10 | 11 | 12 | 13 | 14 | 15 |
| 1. Serve vegetable once a day | 1 | .91, *p*<.001** | .01, *p*=.88 | -.04, *p=.*24 | -.03, *p=.41* | .05, *p=.16* | .02, *p=.45* | .05, *p=.16* | 0, *p=.99* | .06, *p=.06* | .07, *p=.02** | .08, *p=.01** | .05, *p=.16* | .12, *p<.001*** | .03, *p=.38* |
| 2. Prepare vegetable without fat |  | 1 | -.01, *p=.81* | -.01, *p=.69* | -.03, *p=.36* | .04, *p=.27* | .02, *p=.52* | .05, *p=.16* | .03, *p=.38* | .05, *p=.10* | .03, *p=.32* | .07, *p=.03** | .04, *p=.23* | .11, *p<.001*** | .02, *p=.49* |
| 3. Geographical location (Urban=1, Rural=0) |  |  | 1 | -.04, *p=.27* | .32, *p*<.001** | -.28, *p<.001*** | -.29, *p<.001*** | .02, *p=.55* | .04, *p=.19* | 0, *p=.93* | -.04, *p=.53* | -.02, *p=.25* | .02, *p=.55* | -.03, *p=.42* | 0, *p=.94* |
| 4. Participating in CACFP (1=yes, 0=no) |  |  |  | 1 | -.02, *p=.56* | .05, *p=.12* | .01, *p=.74* | -.04, *p=.28* | -.03, *p=.33* | .01, *p=.65* | .12, *p<.001*** | .09, *p<.001*** | .05, *p=.11* | .03, *p=.34* | .03, *p=.29* |
| 5. Food insecurity |  |  |  |  | 1 | -.31, *p*<.001** | .18, *p*<.001** | .03, *p*=.44 | .02, *p=.06* | -.07, *p=.04** | -.07, *p=.03** | -.06, *p=.08* | -.02, *p=.55* | -.11, *p=.001*** | -.03, *p=.29* |
| 6. Food access |  |  |  |  |  | 1 | .37, *p<.001*** | -.03, *p=04* | -.04, *p=.24* | -.04, *p=.23* | .08, *p=.02** | .09, *p=.007** | 0, *p=.91* | .05, *p=.11* | -.01, *p=.80* |
| 7. Child poverty |  |  |  |  |  |  | 1 | -.01, *p=.76* | 0, *p=.94* | -.03, *p=.43* | 0, *p=.78* | .01, *p=.88* | -.05, *p=.13* | -.01, *p=.74* | -.04, *p=.26* |
| 8. Professional development |  |  |  |  |  |  |  | 1 | 0, *p=.97* | -.01, *p=.71* | .02, *p=.62* | -.01, *p=.81* | .01, *p=.76* | -.04, *p=.29* | .06, *p=.06* |
| 9. Role modeling |  |  |  |  |  |  |  |  | 1 | .44, *p<.001*** | .07, *p=.05* | .06, *p=.07* | .11, *p=.001*** | .08, *p=.01** | .13, *p<.001*** |
| 10. Eating same foods |  |  |  |  |  |  |  |  |  | 1 | .07, *p=.03** | .03, *p=.32* | .04, *p=.22* | .14, *p<.001*** | .08, *p=.01** |
| 11.Recommendations |  |  |  |  |  |  |  |  |  |  | 1 | .54, *p<.001*** | .48 *p<.001*** | .51, *p<.001*** | .38, *p<.001*** |
| 12. Lacking time to shop |  |  |  |  |  |  |  |  |  |  |  | 1 | .36 *p<.001*** | .44, *p<.001*** | .4, *p<.001*** |
| 13. Lack of money |  |  |  |  |  |  |  |  |  |  |  |  | 1 | .44, *p<.001*** | .5, *p<.001*** |
| 14. Lacking time to prepare |  |  |  |  |  |  |  |  |  |  |  |  |  | 1 | .4, *p<.001*** |
| 15. Children’s taste |  |  |  |  |  |  |  |  |  |  |  |  |  |  | 1 |
| ***Indicates statistically significant difference at *p < 0.05*, not adjusted for multiple analyses  ****Indicates statistically significant difference at *p<.01*, not adjusted for multiple analyses  ^a^ Child and Adult Care Food Program (CACFP). | | | | | | | | | | | | | | | |
